# Factors affecting online shopping frequency: lessons from New Zealand

**DOI:** 10.1007/s43546-022-00214-5

**Published:** 2022-05-04

**Authors:** Wanglin Ma, Christopher Gan, Puneet Vatsa, Wei Yang, Hongyun Zheng

**Affiliations:** 1 Department of Global Value Chains and Trade, Faculty of Agribusiness and Commerce, Lincoln University, Christchurch, New Zealand; 2 Department of Financial and Business Systems, Faculty of Agribusiness and Commerce, Lincoln University, Christchurch, New Zealand; 3 Present Address: College of Economics and Management, Huazhong Agricultural University, Wuhan, 430070 China

**Keywords:** Online shopping, COVID-19, Poisson regression, New Zealand, B23, C42

## Abstract

During the COVID-19 pandemic lockdown, the number of people shopping online has increased worldwide, and New Zealand is no exception. To date, little is known about the online shopping behaviours of New Zealanders in a pandemic environment. This paper provides the first attempt by exploring the factors affecting online shopping frequency in New Zealand, a country widely regarded as a paragon of excellence for containing the COVID-19 pandemic. A Poisson regression model is utilized to analyze data collected through an online survey between July and November 2020. The empirical results show that people's online shopping frequency is positively affected by payment convenience, competitive pricing, living in the city, and the number of children. The perceived effectiveness of the government's action in combating COVID-19, having poor past online shopping experiences, and being married reduce online shopping frequency.

## Introduction

The COVID-19 pandemic has accelerated the transition to online shopping. Various factors, such as government mandates, lockdowns, physical distancing measures, limits on the number of concurrent customers in retail outlets, fear of contracting COVID-19 virus, and personal precautionary measures, have driven customers to shop online. Chang and Meyerhoefer (2021) found that an additional confirmed case of COVID-19 increases the number of online shopping customers by 4.9% in Taiwan. In India, online food shopping orders from BigBasket and GROFERS, two food e-commerce firms, increased sixfold between March and April 2020 (Reardon et al. 2021). New Zealand Post research shows that New Zealanders spent a total of $5.8 billion shopping online in 2020, which was about $1.2 billion more than in 2019.^1^ More people have adopted online shopping since the outbreak of the COVID-19 pandemic.

A growing number of studies have investigated the factors affecting online shopping of consumers residing in different countries (Doan 2020; Moshrefjavadi et al. 2012; Park and Kim 2003; Putro and Haryanto 2015; SivaKumar and Gunasekaran 2017; Zheng and Ma 2021). Based on a meta-analysis, Ahmed and Sathish (2015) reported that age, gender, income level, and online shopping perception (e.g., perceived behavioural control) are important determinants of online shopping behaviour. A study for Thailand by Changchit et al. (2019) also shows that perceived usefulness, perceived ease of use, and past online shopping experience positively affect consumers' online shopping behaviour. In China, Gao et al. (2020) found that online food shopping during the COVID-19 pandemic is more prevalent among young people living in relatively large cities and having a low perceived risk of online shopping. To be clear, these studies examine the pre-COVID-19 period and thus do not account for the transformative impact of the pandemic on online shopping globally.

New Zealand is widely regarded as a paragon of excellence for containing the COVID-19 pandemic. Three weeks after detecting the first COVID-19 case in New Zealand, its government implemented a 4-tiered Alert Level system on March 21, 2020, to combat the pandemic^2^; its government pursued an elimination strategy. Although the country has moved between the four alert levels and the virus is not eliminated at the time of writing, New Zealanders have enjoyed more freedom during the COVID-19 pandemic than people in other countries. Nevertheless, the pandemic has profoundly affected the New Zealand economy, its residents' mental wellbeing, and their day-to-day lives in general. Furthermore, the pandemic is also changing people’s shopping behaviours and preferences in the country. From panic-buying to online shopping, people are changing what, when, and how they buy. However, their preferences for online shopping could be different due to its sparse population and remote location.

To date, no studies have investigated the factors affecting New Zealanders' online shopping behaviours, particularly during the outbreak of the COVID-19 pandemic. Although the current literature provides insights into the determinants of online shopping (e.g., Ahmed and Sathish 2015; Changchit et al. 2019; Doan 2020; Park and Kim 2003; Putro and Haryanto, 2015), their findings are not directly applicable to New Zealand. For example, due to its small territorial area, sparse population, and remote location, New Zealand has unique supply chains. Moreover, E-commerce penetration in New Zealand remains low relative to China, Germany, and the USA. Nevertheless, E-commerce is proliferating in New Zealand, making it a promising market (EShop World 2019).

This paper is the first attempt to investigate factors affecting New Zealanders' online shopping frequency using the data collected through an online survey between July and November 2020. Considering that this period followed the nationwide lockdown and coincided with Alert Levels 2 and 3, we also analyze how people’s perceptions of the effectiveness of government's measures for combatting the pandemic affect their online shopping behaviour. During Alert Level 2, people are free to travel and participate in events that meet Alert Level 2 requirements. However, they are urged to do so safely. For example, the number of passengers in public transit is limited, and each must be seated. The restrictions on movement and congregations are far more stringent in Alert Level 3—while people may move within their local areas for shopping, exercising, and commuting to work, if necessary, travel is restricted. These restrictions may increase the likelihood of shopping online.

Because we focus on online shopping frequency rather than dichotomous online purchasing decisions of consumers, a Poisson regression model is utilized. As this paper provides actionable insights into the online shopping behaviours of New Zealanders, it should be of strong interest to industry stakeholders such as online retailers and e-commerce platforms seeking to tap into a high-growth promising market.

The rest of the paper is structured as follows: “Methodology” introduces methodology. The empirical results are presented and discussed in “Conclusions”. The final section concludes and proposes implications.

## Methodology

### Data

The paper adopted snowballing sampling approach for data collection. The snowball sampling method is based on referrals from initial respondents to generate additional respondents (Naderifar et al. 2017). Considering that respondents less than 18 years of age may not be independent in making purchasing decisions and thus not provide meaningful answers, we excluded them from the survey. The survey was administered online in New Zealand between July and November 2020. Students and staff members from a university in New Zealand were invited to complete the survey using the online platform (https://jinshuju.net/). Participants were encouraged to forward the survey link to their colleagues, friends, and students. This yielded 176 observations, of which we dropped 23 observations with missing and incomplete information. Thus, the empirical analysis was conducted using 153 observations. An information sheet was attached at the beginning of the survey web page explaining the survey's purpose, process, and length. This was followed by questions regarding the demographic profiles of the respondents. They provided answers using Likert scales and checklists. The University's Human Ethics Committee approved the questionnaire.

Table 1 shows the definitions and descriptive statistics of the variables used in the empirical analysis. The data show that the average online shopping frequency is about 2.9 times per month. Considering the influence of the COVID-19 pandemic on consumers' online shopping behaviour, we ask the respondents about their perceptions of the New Zealand government's success in combatting the pandemic. Next, we determine how these perceptions affect consumers' online shopping frequency. The data show that 88% of the respondents acknowledge the perceived effectiveness of the government's actions against COVID-19 pandemic. Based on the key factors closely related to online shopping, around 41% and 55% of the respondents value payment convenience and the large variety of online shopping products, respectively. Only 13% of them are motivated to purchase online due to the absence of hidden costs. Online pricing is perceived to be relatively competitive, with a mean score of 3.18 (on a scale of 1–5, from not very competitive to very competitive). A total of 39% of the respondents indicate a poor online shopping experience in the past. We include control variables (e.g., age, gender, income level, and work status) in the analysis to control the various demographic characteristics of the sample. Overall, the respondents are young females (aged around 31 years old). Most of them are unmarried, live in a city, and have average incomes falling in the lower-income brackets (45% below NZ$30,000 and 24% between NZ$30,001 and NZ$60,000). Further, 30% of the respondents are religious, and 27% of them work full-time.

**Table 1:** Variable definitions and summary statistics

Variables	Definition	Mean (S.D.)
Outcome variable		
Online shopping	Frequency of online shopping (times/month)	2.89 (3.69)
Key explanatory variables		
Perceived effectiveness	1 = a respondent perceives that New Zealand government's actions in combating the spread of COVID-19 are effective; 0 = otherwise	0.88 (0.33)
Payment convenience	1 = it is convenient to pay online; 0 = otherwise	0.41 (0.49)
Hidden cost	1 = online shopping has no hidden costs; 0 = otherwise	0.13 (0.34)
Product diversity	1 = it is easy to select a wide range of products online; 0 = otherwise	0.55 (0.50)
Price perception	Perception of the competitiveness of online pricing (from 1 = not very competitive to 5 = very competitive)	3.18 (0.99)
Poor past online shopping experience	1 = respondent had a poor online shopping experience (e.g., delay in delivery, poor quality of product, and product damage); 0 = otherwise	0.39 (0.49)
Control variables		
Age	Age of respondent (years)	31.20 (9.86)
Gender	1 = male, 0 = female	0.38 (0.49)
Marital status	1 = married; 0 = otherwise	0.30 (0.46)
Living location	1 = living in a city; 0 = otherwise	0.70 (0.46)
Income_1	Before-tax annual household income (up to NZ$30,000)	0.45 (0.50)
Income_2	Before-tax annual household income (NZ$30,001–60,000)	0.24 (0.43)
Income_3	Before-tax annual household income (NZ$60,001–80,000)	0.14 (0.35)
Income_4	Before-tax annual household income (NZ$80,001–100,000)	0.04 (0.19)
Income_5	Before-tax annual household income (over NZ$100,000)	0.13 (0.33)
Religious	1 = respondent is religious; 0 = otherwise	0.30 (0.46)
Children	Number of children in the respondent’s household	0.52 (1.03)
Full-time work	1 = working as a full-time employee; 0 = otherwise	0.27 (0.45)
Sample size		153

S.D. refers to the standard deviation

### Empirical model

We use a Poisson regression model to analyze the factors affecting online shopping frequency. Poisson regression models the number of occurrences (counts) of an event, and thus, lends itself well to determining online shopping frequency. Specifically, the Poisson regression gives the probability of observing a given value ${y}_{i}$ (the online shopping frequency in our case).

1 $$\Pr \left( {Y = y_{i} | X_{i} } \right) = \frac{{\lambda_{i}^{{y_{i} }} \exp \left( { - \lambda_{i} } \right)}}{{y_{i} !}}, y_{i} = 0,1,2, \ldots ,$$

Here, the observed values of ${y}_{i}$ is drawn from a Poisson population with parameter ${\lambda }_{i}$,

2 $$\lambda_{i} = \exp \left( {\alpha + X_{i}^{^{\prime}} \beta } \right),$$

which is related to the regressors ${X}_{i}$. $\alpha$ is the constant and $\beta$ is a vector of parameters associated with ${X}_{i}$ (Greene 2012). We exponentiate the coefficient estimates of $\beta$ to derive incidence rate ratio (IRR), as the latter indicate how changes in ${X}_{i}$ affect the rate at which ${y}_{i}$ occurs (Colin and Pravin 2013; Ma and Wang 2020).

### Empirical results

Table 2 presents the results obtained from the Poisson regression. Rather than discuss the coefficients (i.e., the change in the expected log of online shopping frequency), we will discuss the IRRs as they are directly interpretable and provide meaningful insights into how different factors influence the frequency of online shopping of New Zealanders. First, let us consider the experiential factors. The results show that shoppers who find online payments easy have a 1.70 times greater online shopping rate relative to those who do not. On the other hand, shoppers who have encountered problems during online shopping have a 0.82 times lower rate of online shopping relative to those who have not. Thus, the positive effect of payment convenience is significantly greater than the negative effect of inconveniences, such as non-delivery, delivery delays, and product spoilage (see Table 3). Interestingly, the absence of hidden costs and the ability to select from a wide range of products do not influence the frequency of online shopping.

**Table 2:** Determinants of online shopping frequency: Poisson model estimation

Variables	Coefficients	IRRs
Perceived effectiveness	− 0.503 (0.133)***	0.605***
Payment convenience	0.512 (0.110)***	1.669***
Hidden cost	0.177 (0.144)	1.194
Products	0.062 (0.113)	1.064
Price perception	0.096 (0.054)*	1.101*
Poor past online shopping experience	− 0.193 (0.111)*	0.824*
Age	0.008 (0.006)	1.008
Gender	− 0.066 (0.103)	0.936
Marital status	− 0.259 (0.119)**	0.772**
Living location	0.712 (0.131)***	2.039***
Income_2	0.230 (0.126)*	1.259*
Income_3	− 0.023 (0.176)	0.977
Income_4	0.212 (0.253)	1.236
Income_5	− 0.799 (0.221)***	0.450***
Religious	0.032 (0.127)	1.032
Children	0.163 (0.049)***	1.177***
Full-time work	0.081 (0.126)	1.084
Constant	0.098 (0.312)	1.103
Sample size	153	

* < 0.10, ** < 0.05, and *** < 0.01. Standard errors are presented in parentheses. The reference income group is Income_1

**Table 3:** Problems facing online shoppers

Category	Frequency	Per cent of responses
Delay in delivery	43	69.35
Product damage	10	16.13
Non-delivery	9	14.52

Unsurprisingly, price perception significantly influences consumers' online shopping behaviour. Specifically, perceiving prices to be more competitive—i.e., a one-step increase on a five-point Likert scale—is associated with 1.10 times greater online shopping rate. The COVID-19 pandemic has accelerated the shift to online shopping globally. Fear, caution, government mandates, and lockdown have prevented people from visiting shopping centres. In New Zealand, the level of trust in the government's handling of the COVID-19 pandemic has been remarkably high (Wilson 2020); according to an IPSOS (2021) report, 83% of New Zealanders believe that the government has done well to contain the pandemic. We find that people's beliefs regarding the government's actions to contain the pandemic significantly affect online shopping. The online shopping rate amongst those who believe, relative to those who do not, that the government's actions effectively combat the pandemic is 0.61 times smaller. This suggests that effective containment policies spur in-person shopping while negatively affecting online shopping. These results are consistent with a recent study by Alhaimer (2022), who reports that government mandates and penalties to contain the COVID-19 pandemic in Kuwait positively affect consumer behaviour towards online shopping.

Lastly, we consider the effects of demographic and location factors. Which categories of customers have higher online shopping rates? The answer to this question can inform customer segmentation and targeted marketing initiatives to enhance sales. We find that although being married reduces the online shopping rate, having children increases it. As such, compared to unmarried people, married people have a 0.77 times smaller online shopping rate; however, those with children have a 1.18 times greater online shopping rate than those without children. Many couples spend a considerable amount of time apart during the week due to their work commitments. Shopping in physical stores allows married couples to spend more time with each other. Although trips to the grocery store are somewhat mundane activities, married couples may view these as excursions. In doing so, they make meal preparation a more immersive and collaborative experience. However, shopping with children may present some challenges. Children can behave uncooperatively, making it unenjoyable and stressful to shop in physical stores (Naseri and Elliott 2011). To avoid such difficulties, couples with children may choose to shop online instead. Further, married couples and larger households are more likely to involve children and, as such, time becomes more valuable and the cost of conventional shopping increases.

Unlike previous research that finds a positive link between income and online shopping (see, for example, Zhou et al. 2007), we show that the effect of income on online shopping rate is not monotonic. Although income positively affects online shopping frequency at lower income brackets, it negatively affects the same at higher income brackets. For example, people earning between NZ$30,001–60,000 have 1.26 times the online shopping rate than people earning less than NZ$30,001; however, people earning more than NZ$100,000 have a markedly lower online shopping rate (i.e., 0.45 times) compared to those in the lowest income bracket. The data also show that location exerts significant influence over online shopping frequency. People living in cities have twice the online shopping rate compared to non-city dwellers. Shipping and delivery to rural and remote areas entail relatively higher costs. Furthermore, some retailers do not deliver to such areas. Underdeveloped local delivery systems may lower online shopping rates amongst rural and remote dwellers (Song 2021).

## Conclusions

Online shopping surged in New Zealand during the outbreak of the COVID-19 pandemic in 2020. This paper makes the first attempt by investigating the factors influencing New Zealanders' online shopping frequency. Using a Poisson model, the paper discloses that payment convenience, competitive pricing, living in the city, and the number of children significantly and positively affect online shopping frequency. On the other hand, the perceived effectiveness of the government's action in combating COVID-19 pandemic, poor past online shopping experience, and being married reduces online shopping frequency. These findings may inform market segmentation, promotion initiatives, and e-commerce strategies in New Zealand. In particular, if online marketers and retailers can better understand their customers’ shopping behaviour or preferences, they can present goods and services more effectively to improve their offerings to strengthen their competitive advantage. Moreover, they suggest that the measures taken by the government during the pandemic strongly influence people's shopping preferences. These results also underscore the importance of ensuring that customers have a positive online shopping experience. This will generate repeat visits to online shopping platforms and increase the likelihood of acquiring new customers through positive word-of-mouth.

We recognize that the sampling technique used in this paper precludes broad generalizations of these results. Whether the momentum in online shopping growth will be sustained after the pandemic remains an open question. Furthermore, it would be interesting to analyze how these results generalize to shopping behaviours globally. We suggest that future research be devoted to answering these questions as more data become available over time. For example, future researchers can undertake comparative studies to investigate the shopping preferences of consumers from different countries, such as New Zealand and Australia, as well as explore consumers’ shopping preferences pre and post Covid-19 pandemic. Also, focussing on specific shopping categories (e.g., goods versus services or luxuries versus necessities) may offer additional and actionable insights to marketers.

## Data Availability

The data and material supporting this study’s findings are available from the last author, Hongyun Zheng, upon reasonable request.

## Footnotes

1 https://www.nzpost.co.nz/about-us/media-centre/media-release/nz-post-research-shows-kiwis-spent-over-a-billion-dollars-more-online-in-2020-than-in-2019.

2 Introduction of the Alert system in New Zealand is available at: https://covid19.govt.nz/alert-levels-and-updates/about-the-alert-system/.
